# President's speech at 34^th^ Annual Conference of Indian Association of Pediatric Surgeons, Guwahati

**DOI:** 10.4103/0971-9261.54808

**Published:** 2009

**Authors:** Anirudh Shah

**Affiliations:** President, Indian Association of Pediatric Surgeons, Amardeep Multispeciality Children Hospital and Research Centre, Ahmedabad, India E-mail: dranirudhshah@gmail.com

Honorable Shri U. C. Sharma, Director of Medical Education, Government of Assam, and chief guest of the evening; Shri M. M. Deka, Principal, Guwahati Medical College, and guest of honor; Dr. H. K. Borah, Chairman, organizing committee; Dr. Nirmal Bhattacharyya, Organizing Secretary; members of the executive committee; past Presidents and Secretaries of this association; invited guests; faculty; delegates; and ladies and gentlemen.

I feel honored to be here as the President of our august association on the inaugural function of the 34^th^ annual conference at this beautiful city of Guwahati. The terrorist attack of October 30^th^ and its aftermath forced us to postpone our meeting by a month. Such cowardly acts of terrorism shock us, but do not deter our sprits and commitment toward love, justice, mercy, and working towards the progress of our country and our specialty.

As I stand here talking before you, I still remember the mid-70's when I returned back to Ahmedabad after my pediatric surgical training in the United Kingdom. I started pediatric surgery in Gujarat with four beds in a busy 1000-bedded Municipal Hospital in Ahmedabad. We have progressed from those days when vascular access used to be the first and major challenge for any neonatal or pediatric surgical problem to advanced neonatal and pediatric minimally invasive surgery. Today, I can proudly state that many centers in India including Ahmedabad are doing a huge amount of excellent work which is at par with many leading centers across the world.

It has been almost 53 years since IAPS branched out from general surgeons as a separate group of people who were not only interested but also dedicated to deal with surgical problems of children. We are in our 15^th^ year as a separate association. As is characteristic of Indians, we have made a lot of progress in not only on the national but also on the international scene. We have now reached a stage where we can put forward our views and experiences with confidence and authenticity and can be sure that pediatric surgeons across the globe listen to us with conviction.

However friends, it is still too early to pat our backs at this point of time. India won a much awaited individual gold medal and two bronze medals at the recent Beijing Olympics, but countries like Ethiopia, Turkey, Uzbekistan, and Zimbabwe have been winning more medals than us for years. As per UNICEF 2007 records, India registers the highest number of child deaths across the globe. Of the estimated 9.7 million children in the world dying before completing five years of age, 21% (i.e. 2.1 million) are in India. Also, 50% of child mortality is due to neonatal reasons, as opposed to 37% across the world. As we all know, neonatal anomalies account for almost 10% of the total neonatal mortality rates and this is where we pediatric surgeons would be playing a major role. There are still many pediatric surgical centers across India which are managing a phenomenal amount of pediatric surgical workload with meager resources, lack of trained manpower, and poor backup facilities leading to a high morbidity and mortality. Training of paramedical personnel, nurses, and technical staff should be made priority if we want to come out with good pediatric surgical results.

Female feticide though legally banned in India is not so successful. Feticide should be legally banned but it should not be a sex-biased law. Lots of male fetuses are also aborted for minor defects which are easily correctable postnatally in India. Is this legally permissible? I want that we should raise our voice of banning feticide as a whole and not a sex-biased law.

Over the past year, there has been a lot of exchange of knowledge and clinical experience in the form of CME's, seminars, conferences, and workshops across the country. Details of the same are there in the Secretary's report and I will avoid repetition of the same. My congratulations to all the organizers. Over and above all these meetings, many state chapters and subchapters of IAPS have been diligent and have carried out their academic activities with precision. Special congrats to the Tamilnadu and Pondicherry chapter of IAPS, headed by Prof. Sathappan and Dr. Raveenthiran, which organized its seventh annual conference and preconference workshop at Vellore in July. Quality of workshop organization was taken to a higher level by Dr. Srimurthy, Dr. Ramesh, and their team at Bangalore when they organized the International Workshop on Advanced Pediatric Laparoscopy in August. The urodynamics workshops organized by Pediatric Urology subchapter of IAPS across different centers of the country have also been generating a lot of interest. I request other subchapters of IAPS to catch up.

We are proud that our fellow member and past President Dr. D. K. Gupta is presently the President of the Asian Association of Pediatric Surgeons (AAPS) and also President of Federation of Association of Pediatric Surgeons of SAARC (FAPSS). He has also been given the responsibility to organize the World Congress of Pediatric Surgeons in 2010 at Delhi. I had the honor and privilege to meet Prof. P. Upadhyaya, the recipient of this year's R. K. Gandhi gold medal, at a meeting organized to honor him on his 80^th^ birthday at New Delhi. I felt proud to see the amount of respect this great stalwart received from senior colleagues from across the world who had come all the way specially to greet him.

Honorary member of IAPS Prof. Prem Puri has been elected as the President of the World Federation of Associations of Pediatric Surgeons (WOFAPS). He also received Honorary Fellowship from the American Academy of Pediatrics for his work on VUR.

The year gone by has been shocking for our association in that we lost a dear friend Dr. K. K. Sharma. This is the first time in the history of our association that we have faced such a calamity. Dr. Sharma was a dynamic and efficient person. He had set himself high goals for the betterment and progress of IAPS during his tenure as Secretary cum Treasurer. As a tribute to the departed soul, we have named the session on GI surgery at this conference on his name. Dr. V. Sripathi needs special mention for the way in which he has shouldered the responsibility of Secretary cum Treasurer after the sudden demise of Dr. Sharma. He has been immaculate, prompt, and also very considerate in carrying out his duties. A special word of thanks to Venkat!

I would like to sincerely thank Dr. H. K. Borah, Dr. N. C. Bhattacharyya, and the entire team of IAPSCON 2008 for their dedicated hard work to put up a fantastic show at Guwahati in spite of all the odds. This year's IAPSCON is unique for it is a 'complete Indian affair'. All our CME speakers, orators, paper presenters, and even delegates are Indians. I am sure you all will agree that they have put up an excellent show. The next IAPSCON will be held in the historic city of Lucknow. Dr. S. N. Kureel, Dr. R. K. Tandon, Dr. Ashish Wakhlu and their team will leave no stone unturned to make it a memorable event for you all.

I would like to thank all my friends and seniors who have helped me in the times of need during my tenure as President of this august association. Thanks to all my executive committee members and past President Dr. Biswanath Mukhopadhyay for their support. I now pass the baton to my successor Dr. V. K. Raina who I am sure will take the association to further heights. Finally, I would like to thank my wife Nita and my son Dr. Amar Shah who have been a constant source of inspiration and whole-hearted support.

Thank you all for giving me this opportunity to serve you all as President, IAPS.

Wishing you all a happy and fruitful time in Guwahati

Long live IAPS

Jai Hind

